# Application of wide-field infrared reflectance imaging in retinoschisis, retinal detachments, and schisis detachments

**DOI:** 10.1186/s40942-019-0188-5

**Published:** 2019-12-12

**Authors:** Himanshu K. Banda, Anjali Shah, Gaurav K. Shah

**Affiliations:** The Retina Institute–St. Louis, 1600 S. Brentwood Blvd Suite 800, St. Louis, MO 63144 USA

**Keywords:** Wide-field infrared imaging, Retinoschisis, Retinal detachment, Retinoschisis detachment

## Abstract

**Background:**

Retinoschisis and retinal detachment are distinguished based on features in clinical examination. Even to skilled examiners, some cases may be diagnostic challenges. Infrared and wide-angle infrared reflectance imaging are relatively new modalities that can provide additional diagnostic information. Non-contact infrared reflectance imaging (also described as near-infrared imaging) highlights sub-retinal features which may otherwise be obscured by standard retinal photography. It is non-invasive and uses the retina’s ability to absorb, reflect or scatter infrared light to produce high quality images.

**Main body:**

The aim of this review is to describe the role of wide-field infrared imaging in screening, diagnosing, and monitoring structural peripheral retinal disorders including retinoschisis, retinal detachment or combined retinoschisis rhegmatogenous detachments. Infrared imaging can also be used to monitor anterior segment inflammation. Heidelberg Wide-Field Module lens and Heidelberg Spectralis^®^ HRA + OCT machine (Heidelberg Engineering, Heidelberg, Germany) were used to obtain noncontact, wide-field infrared images on each study eye. Pseudocolor photos were captured by Optos Optomap^®^ (Optos, Inc, Massachusetts, USA).

**Conclusion:**

Wide angle infrared imaging offers a quick, noncontact, and noninvasive way to help specialists accurately diagnose, monitor for progression, and educate patients about retinal detachment, retinoschisis and even anterior segment inflammation.

## Background

Retinoschisis (RS), also known as degenerative RS or acquired RS, is a peripheral retinal lesion with a reported prevalence ranging from 1.65% [1, 2] to 7% [3] among persons greater than 40 years old. RS is defined as “as split in the neurosensory retina,” with typical RS occurring in the outer plexiform layer [4]. Typical RS presents as smooth, translucent bullous elevations with immobile fluid that does not collapse with scleral depression. Peripheral microcystoid degeneration of the inner and/or outer retinal holes may be present [5]. Alterations of the retinal pigment epithelium (RPE) and hemorrhages are usually absent [6]. The scotoma created by schisis cavities tends to be absolute, and RS tends to have a reaction to laser. Laser photocoagulation is based on the retinal blanching effect of photocoagulation, which occurs when the neurosensory retina is attached to the RPE.

Retinal detachments (RD) may have variable features on fundus examination. They are usually bullous with corrugations on the surface. Unlike RS, RD may reveal RPE changes such as demarcation lines and evidence of proliferative vitreoretinopathy (PVR) in chronic cases. RD may have variable shifting fluid, therefore allowing indentation by scleral depression. The scotoma created by fluid from an RD tends to be relative. When detached retina with subretinal fluid (SRF) is treated with laser, usually no or minimal reaction is noted.

Other than the fundoscopic findings describe above, there are testing modalities that can be used to help differentiate RS from RD. Optical coherence tomography (OCT) has proved to be useful in distinguishing the two entities [4]. OCT can be performed on the peripheral retina, and in typical subtypes, splitting of layers of the outer and inner plexiform layers can be demonstrated. However, artefacts (mirror artefact) and difficulty imaging the peripheral retina (positioning, fixation, etc.) can make capturing OCT images challenging. Perimetry theoretically may be of use in differentiating between RS and RRD as areas of peripheral retina with RS create an absolute scotoma. However, RS is often located anterior to the equator, and visual defects may not be detected with automated perimetry [7].

Differentiating between RS and RD is important as management and treatment can be drastically different. Cases of non-progressive asymptomatic RS are often observed if they do not involve central vision. Retinal detachment typically require repair in the office or operating room. Despite information from history, examination, OCT and perimetry, differentiating between RS and RD can be a diagnostic challenge even to skilled examiners. One technique described in the literature that can be useful in determining RS and RD is infrared imaging [5]. Wide-field infrared imaging as a quick, noncontact, noninvasive method to accurately diagnose and monitor progression of RS, RD, or combined retinoschisis rhegmatogenous detachments (RS/RD).

## Principles of IR imaging

The ocular fundus contains a variety of substance that absorb, reflect and scatter infrared light. Infrared (IR) reflectance imaging (also described as near-infrared imaging) uses the variability of such reflectance patterns to highlight structures in the sub-retinal space. To acquire IR images, an IR light source (wavelength of 820 nm) coupled with a confocal scanning laser ophthalmoscope (SLO) directly scans the fundus. An en face image is created—the extent of light scatter from layers other than the point of illumination is governed by the aperture of the light source. The primary fundus molecules that absorb infrared light include oxygenated hemoglobin, hemoglobin and water. Melanin, however, is a strong reflector and has less absorption than visible wavelengths.

In clinical applications, IR reflectance imaging highlights sub-retinal features and pathology by penetrating further through the fundus than other modalities [8]. In the pre-OCT era, visualization of sub-retinal structures was critical in clinical practice. IR imaging has the capability to detect drusen which is otherwise gone undetected by ophthalmic examination or color photography. It also had a role in detecting choroidal neovascular membranes non-invasively when compared to fluorescein angiography [9]. Abnormalities on IR imaging may be classified as increased (hyperreflective), reduced or absent (hyporeflective).

## Retinal detachment

**Fig. 1:**
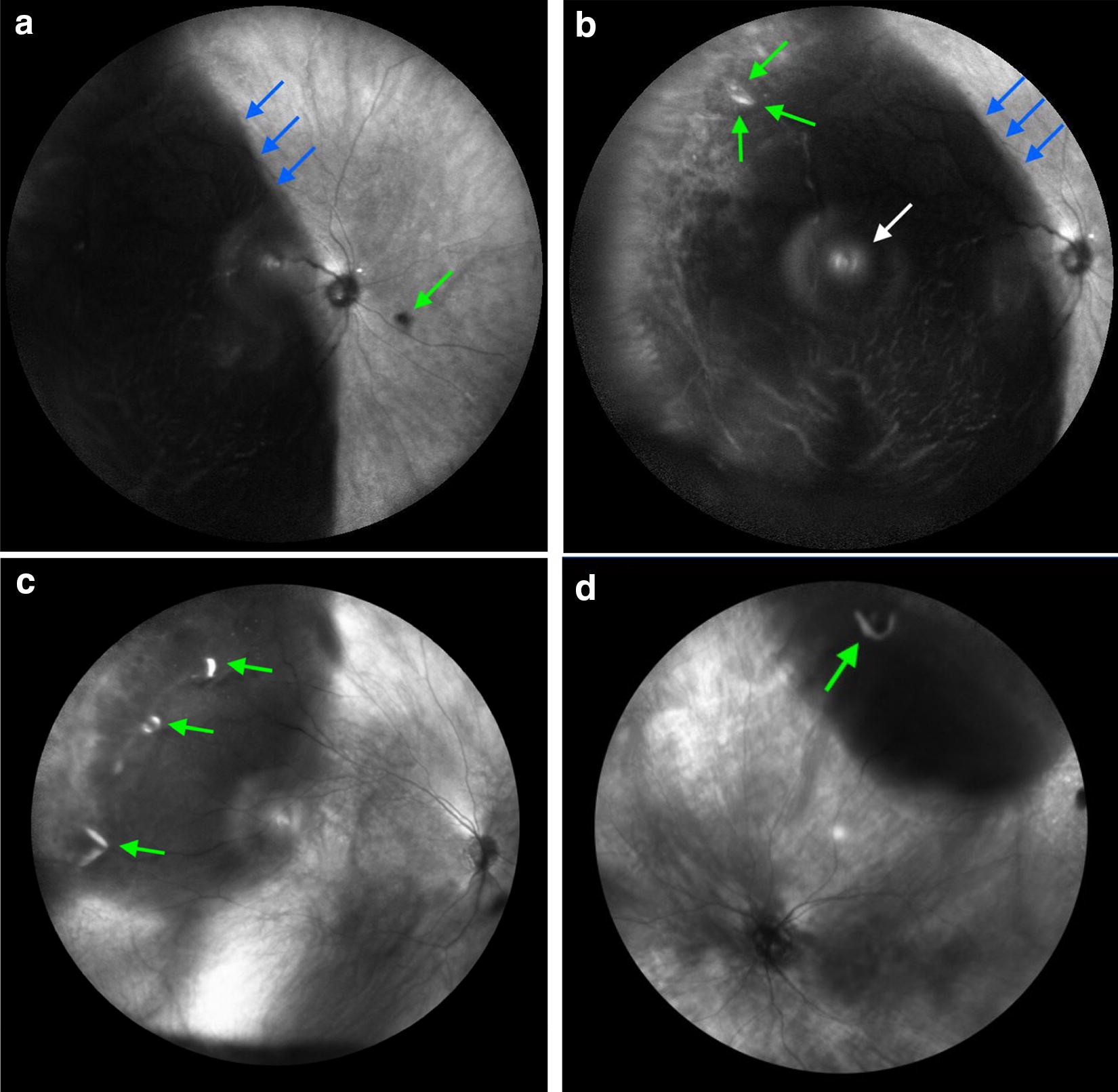
**a** Demonstrates a wide-angle infrared imaging of a temporal macula involving retinal detachment (blue arrows) which appears hyper-reflective. The green arrow reveals hyper-reflective Weiss ring. Witch shifting the wide-field lens to a nasal view (**b**) a retinal tear (green arrows), as well as a reflection artifact (white arrow) can be seen. **c**, **d** Other examples of a retinal detachment with retinal defects defects (green arrows)

IR reflectance signaling is reduced (hyporeflective) in the presence of subretinal fluid or blood due to light scatter [10]. Fluid from retinal detachments tend to appear dark and opaque. Retinal tears may be hyper-reflective on IR as the underlying RPE is exposed. Figure 1a demonstrates a temporal macula involving (mac-off) RD with fluid extending from approximately 5:30 to 11. The detachment is bullous with increased hyporeflectivity. Figure 1b demonstrates the tear which appears hyporeflective. Figure 1c shows a retinal detachment with 3 discrete hyporeflective breaks along the vitreous base. Figure 1d as another retinal detachment with a single retinal tear.

**Fig. 2:**
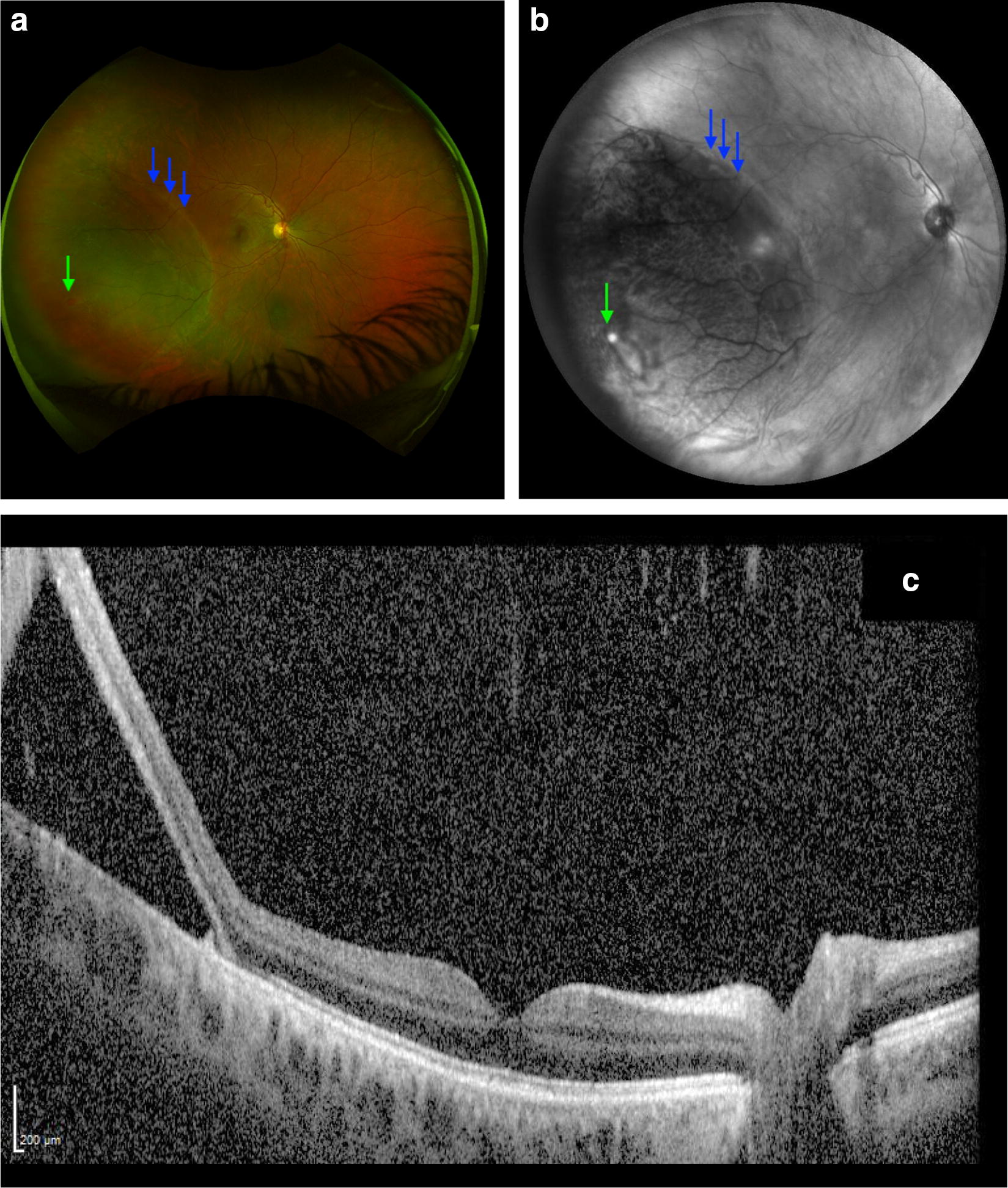
**a** Demonstrates a wide angle color photo inferotemporal retinal detachment (blue arrows) of the right eye with causative break (green arrow). Wide angle infrared image (**b**) demonstrates a hyperreflective tear (green arrow) and associated hyporeflective detachment (blue arrows). **c** Is an OCT through the detachment demonstrating subretinal fluid beneath all retinal layers

Figure 2a demonstrates a wide angle pseudocolor photo (Optos Optomap^®^) inferotemporal retinal detachment of the right eye. Wide angle IR image (Fig. 2b) demonstrates a hyperreflective tear and associated hyporeflective detachment. Compared to the Fig. 1, the RD in Fig. 2b is less hyporeflective as the RD is more shallow. Figure 2c is an OCT through the detachment demonstrating subretinal fluid beneath all retinal layers.

## Retinoschisis

**Fig. 3:**
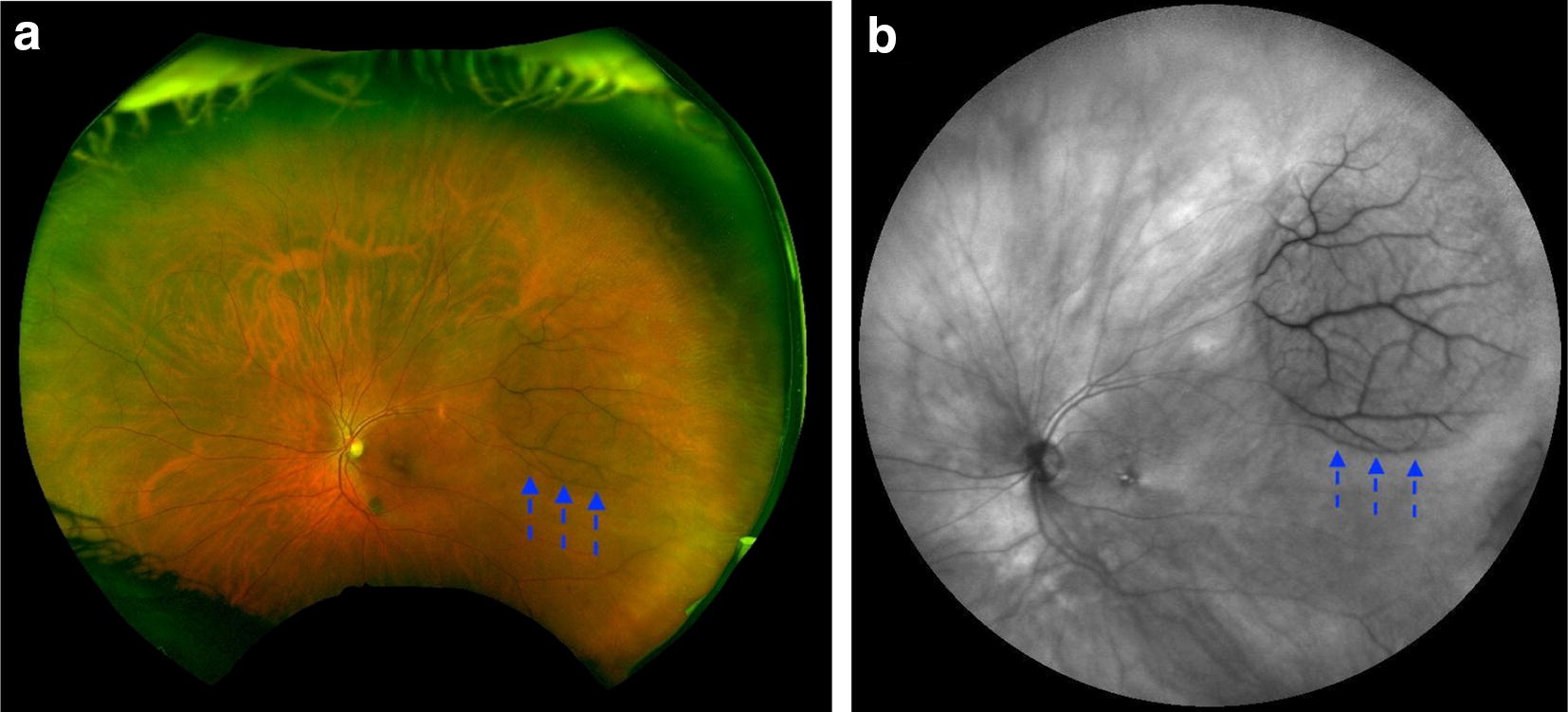
**a** Shows a well-circumscribed retinoschisis cavity in the peripheral retina extending anteriorly (hashed blue arrows). **b** Is a wide angle IR image of the same peripheral RS cavity which appears isoreflective

**Fig. 4:**
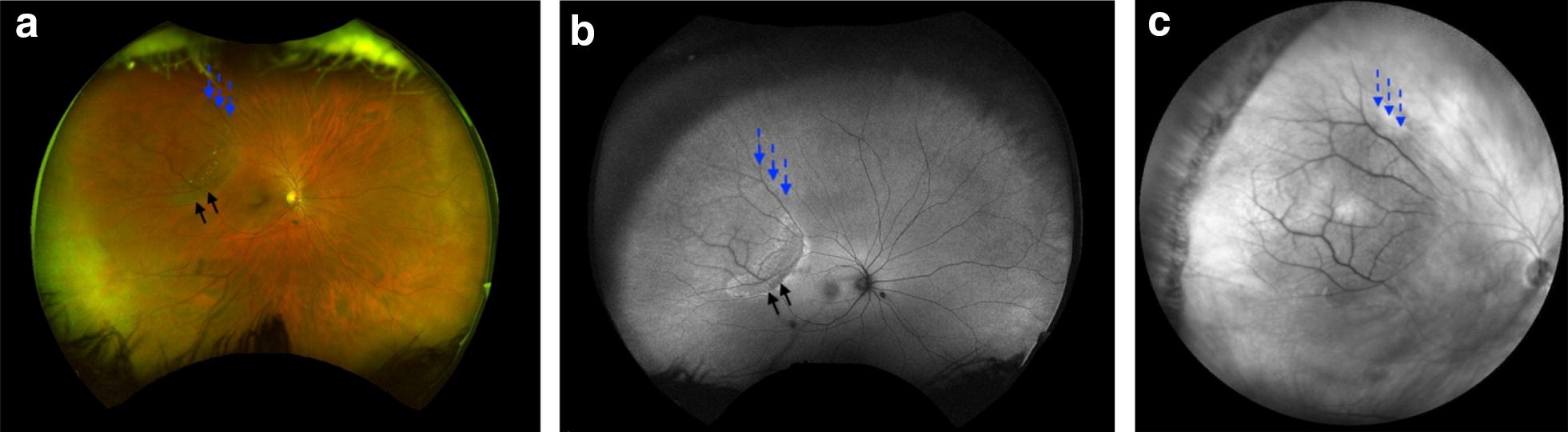
**a** Is a wide-angle color photo of a schisis caivity (line blue arrows) with RPE changes at the edge of the schisis cavity (black arrows). **b** Demonstrates RPE alterations with autofluourescence.** c** Outlines the schisis cavity with wide-angle IR imaging (hashed blue arrows)

**Fig. 5:**
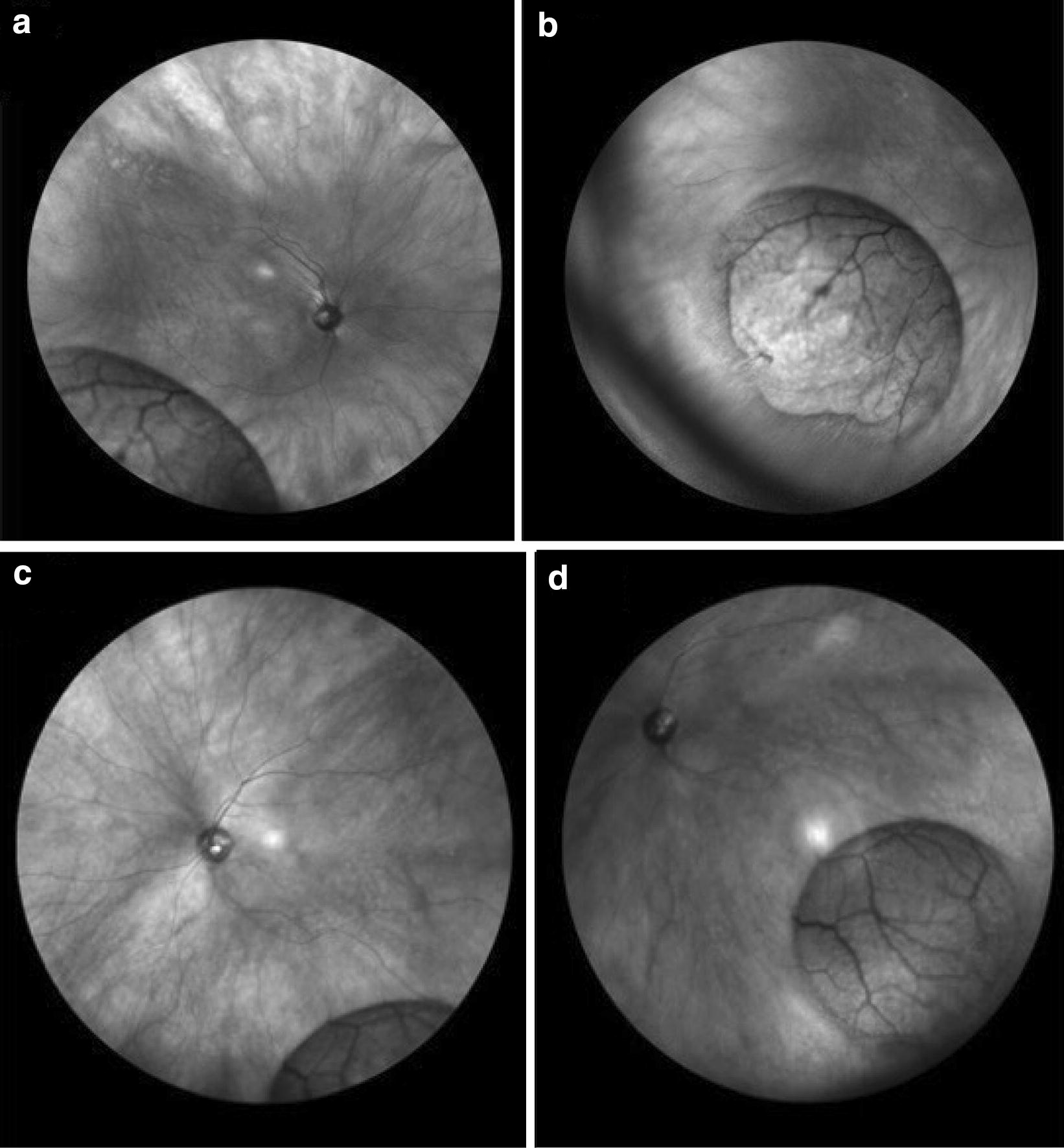
Schisis cavities noted in right (**a**, **b**) and left (**c**, **d**) eyes in inferotemporal quadrants

On color photography, peripheral RS appears classically as dome-shaped translucent elevations prominent vasculature. RS is typically located in the temporal quadrants however can be seen anywhere in the retina [11]. Figure 3a shows a well-circumscribed RS cavity in the peripheral retina extending anteriorly. The vessels are slightly blurred because of the elevated nature of the RS extending out of the focal plane of the camera. Figure 3b is a wide angle IR image of a peripheral RS cavity that appears isoreflective. Figure 4a is a wide-angle pseudocolor photo of the fellow eye demonstrating RPE changes at the edge of the schisis cavity. RPE alterations best demonstrated by autofluorescence (Fig. 4b) while the cavity is highlighted by IR (Fig. 4c). Though ‘demarcation lines’ are not typical for RS, RPE changes may represent regression or collapse of schisis cavity. Figure 5 demonstrates a more classic finding of peripheral RS in the inferotemporal quadrants of a right (a) and left (b) eye.

## Retinoschisis detachment

**Fig. 6:**
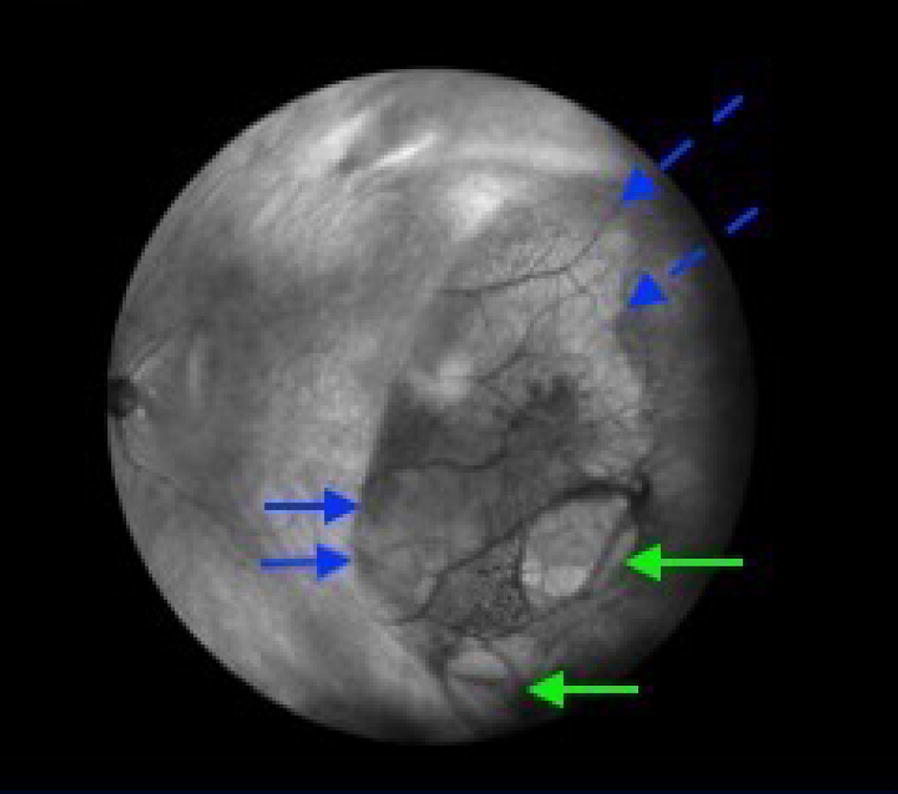
Wide-field IR of a retinoschisis retinal detachment (RS/RD). Green arrows indicate the outer retinal defects, while solid blue lines show the SRF. The schisis cavity is superior to the outer defects noted in hashed blue lines

IR imaging may also be useful in defining retinoschisis detachments (RS/RD). Figure 6 shows an retinal detachment with SRF extending toward the macula. In the image, SRF is hyperreflective and the outer retinal hole appears hyporeflective. Superior to the outer retinal hole, a schisis cavity is noted.

## Widefield IR in monitoring RT and RD

**Fig. 7:**
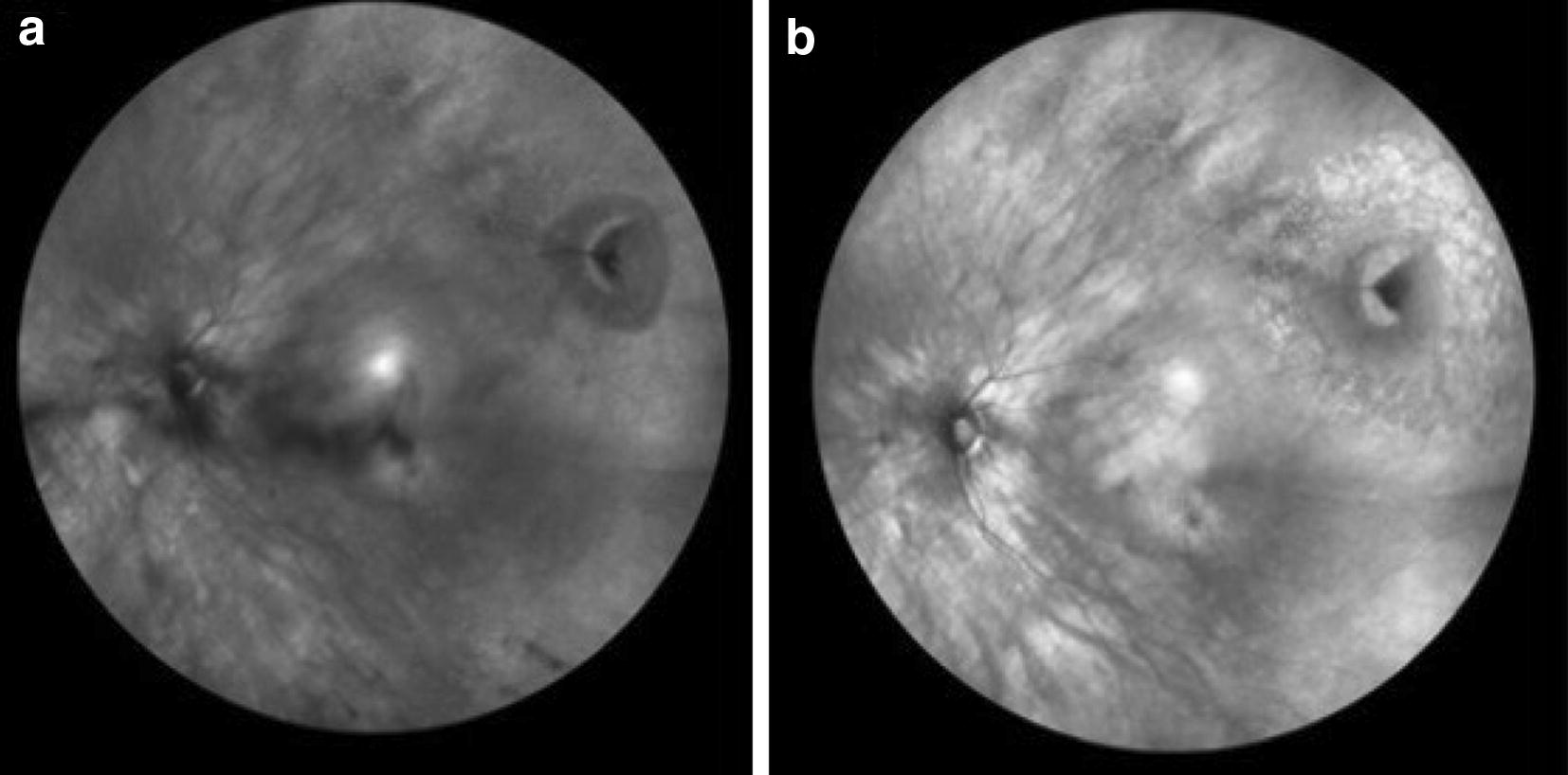
Retinal tear with associated SRF which appears hyperreflective (**a**); tear status post laser retinopexy (**b**). Note hyporeflective laser demarcation likely representing reflection of RPE

**Fig. 8:**
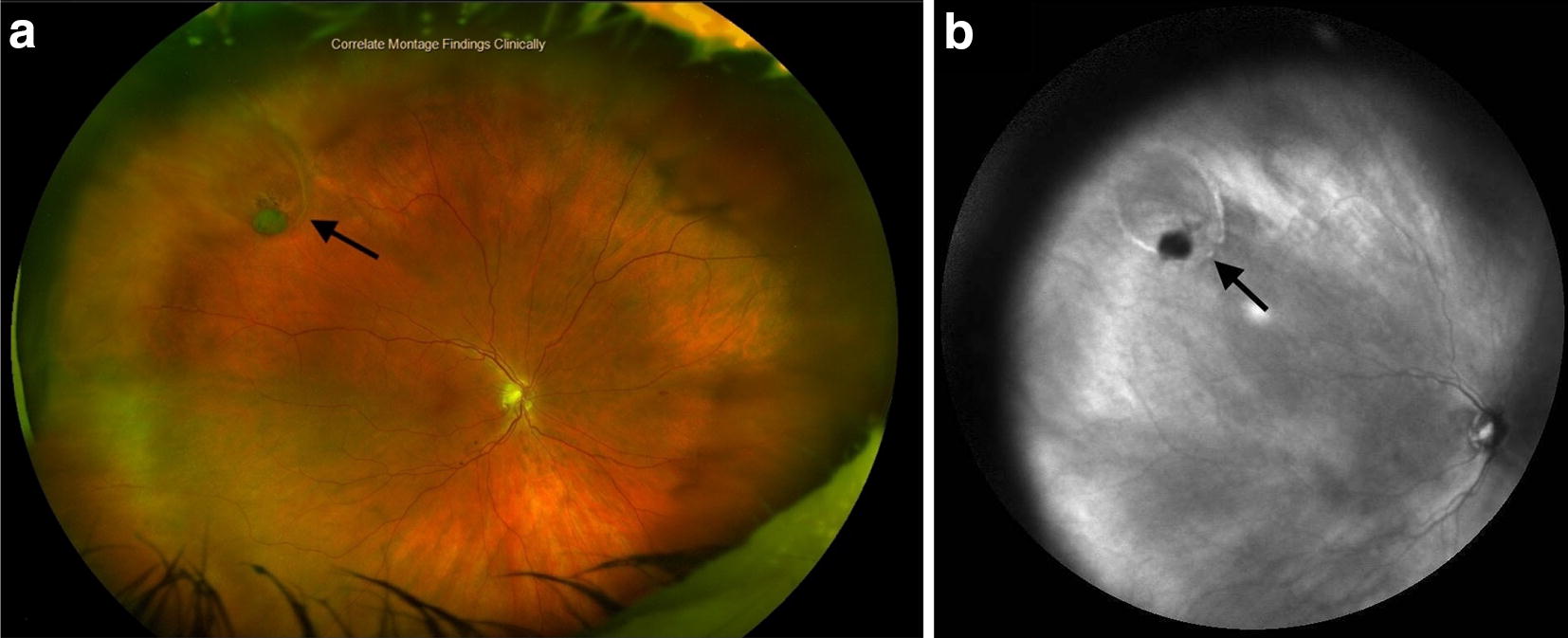
Retinal tear (black arrow) seen in wide field color imaging (**a**) with corresponding wide-field IR (**b**). In IR image, outline of tear appears hyporeflective with a hyperreflective media opacity (vitreous hemorrhage). Compared to Fig. 7a, note lack of hyperreflective SRF in this example

**Fig. 9:**
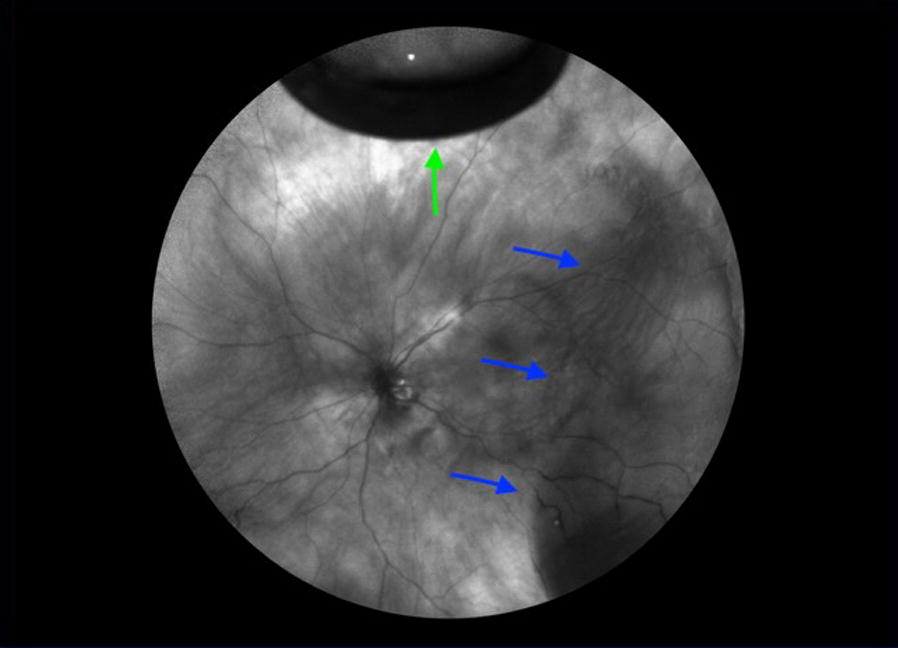
Wide field IR of a retinal detachment status post in-office pneumatic retinopexy. Gas bubble (green arrow) is sealing superior break. Resolving SRF can be noted (blue arrows)

Wide field IR imaging can be used to follow resolution or progression of subretinal fluid. Figure 7 demonstrates a retinal tear with a cuff of hyperreflective SRF (a). After laser demarcation, treatment appears hyporeflective, likely due to reflection of RPE after photocoagulation (b). An IR image is taken of another retinal tear (Fig. 8) demonstrating the absence of SRF, or hyperreflective cuff. Figure 9 demonstrates an after an in-office pneumatic retinopexy of a retinal detachment. Again, residual SRF is noted as hyperreflective.

## Other unique applications of IR imaging

Infrared reflectance imaging has many uses to further describe the choroid an retina, however there are understated applications in anterior segment pathology. Infrared reflectance imaging can be an informative tool in following anterior segment inflammation.

There are many methods in use to quantify and qualify intraocular inflammation. The Standardization of Uveitis Nomenclature (SUN) Working Group created grading schema for anterior segment cell and flare based off observation under slit-lamp biomicroscopy [12]. Other methods exists to assess anterior segment inflammation including commercially available flare meters [13]. Corneal opacities such as edema may hinder observation of the anterior chamber to assess inflammation. IR wavelengths, compared to visible light, are not subject to scatter and can penetrate such corneal opacification [14].

**Fig. 10:**
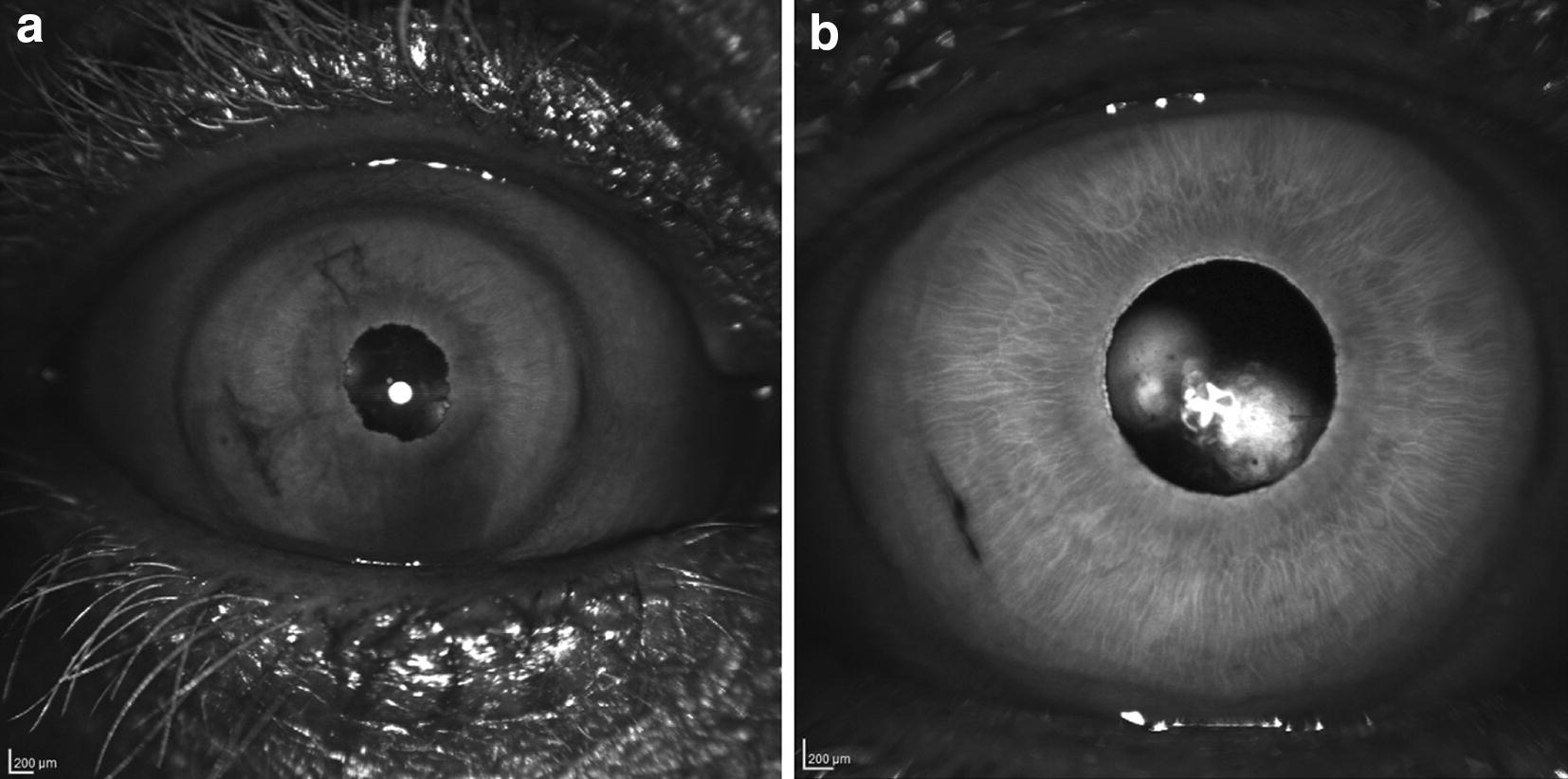
External IR reflectance images of the anterior segment of a patient presenting with post-operative endophthalmitis following cataract surgery. **a** reveals hyperreflective fibrin strands at the incision at the incision as well as hyperreflective debris layered on the endothelium (in the configuration of Arlt’s triangle). After successful treatment (**b**), the hyperreflective fibrin strand is nearly resolved, and iris sturctures appear more hyporeflective indicating decreased cellular debris in the aqueous humor

Inflammatory debris in the anterior chamber in the form of cell, flare or fibrin can be visualized with anterior segment IR. Figure 10a demonstrates a case of post-operative endophthalmitis following cataract surgery. Slit-lamp examination revealed an edematous cornea with a seidel positive incision. IR imaging is able to penetrate through the corneal edema and reveal a fibrin strands at the edge of the clear-corneal wound and paracentesis incisions. Layered cellular debris is noted to be hyperreflective in the configuration of Arlt’s triangle. Figure 10b depicts an IR image of the anterior segment of the same patient following treatment with intravitreal antibiotics. The hyperreflective fibrin bands appear attenuated, and iris details appear more hyporeflective, suggesting resolution of cellular debris in the anterior chamber.

## Conclusions

Diagnosing and monitoring RS, RD, RS/RD can be challenging. Clinical clues and imaging techniques are used to differentiate between these entities. We add to the literature additional examples of wide-field IR images in these conditions.

RDs appear dark and opaque (Fig. 1a), while RS appears light and translucent with prominent vasculature (Fig. 2b) and combined RS/RD exhibit mixed reflectivity patterns. Retinal tears appear hyperreflective (Fig. 6). The isoreflective properties of the retina are restored after successful repair of RDs in this study and fluid cavities related to other retinal pathology in literature studies [15].

Advantages of IR imaging include the ability to capture nonmydriatic photos, ease of use in patients unable to fixate, use of low light levels for photophobic patients, and maintenance of quality images relatively independent of media changes such as cataracts, clouded lens capsules, or mild vitreous hemorrhage/debris [16, 17]. Furthermore, wide-field IR imaging is faster (approximately 5 min) and requires fewer photographs than traditional montage fundus images, which assemble 30 to 60, 50° images over the course of 10 to 30 min [5].

From a monitoring standpoint, wide-field IR can be useful. In addition to standard fundus photography, serial IR imaging may be an important adjunct to detecting outer retinal pathology with patients with peripheral retinoschisis. Additionally, it can be used to detect presence or progression of subretinal fluid surrounding retinal tears or detachment after treatment (Figs. 7 and 9).

There are limitations to wide-field IR imaging. Investment in a wide-field lens system (roughly 20,000 US Dollars for 102° view Heidelberg Wide-Field Module lens) may be cost-prohibitive. However, many practices may have access to this as wide-field imaging in equipment for color photography and fluorescein/indocyanine green angiograph has already been integrated. Additionally, there are photographic artifacts that can occur with IR imaging. The central reflection of the lens can obscure underlying retinal pathology (Fig. 1a). This artifact can be reduced by rotating the patient’s head a few degrees or changing the central fixation location.

Wide-field IR imaging can identify retinal breaks and defects in detached retina, however there is insufficient evidence supporting it as an effective unimodal method for diagnosis of RS, RD, RS/RD [5]. Wide-field IR imaging is best used as a supplement to existing imaging modalities and examination techniques. This novel technique offers a quick, noncontact, and noninvasive way to help specialists accurately diagnose, monitor for progression, and educate patients about these entities.

## Data Availability

All images are original and unpublished, provided upon request.

## Abbreviations

IR: infrared
RS: retinoschisis
RD: retinal detachment
RPE: retinal pigment epithelium
PVR: proliferative vitreoretinopathy
SRF: subretinal fluid
OCT: optical coherence tomography
